# Effectiveness of oregano essential oil vapor on shelf life extension of kai lan (*Brassica oleracea var. alboglabra*)

**DOI:** 10.1111/1750-3841.17673

**Published:** 2025-01-19

**Authors:** Weichen Shu, Zhuoliang Deng, Lingdai Liu, Jiaxuan Zhang, Dan Li

**Affiliations:** ^1^ Department of Food Science and Technology National University of Singapore Singapore Singapore

**Keywords:** antibacterial, antifungal, green color protection, kai lan, oregano essential oil vapor

## Abstract

This study compared the antimicrobial activity of several essential oils (EOs) vapor against food spoilage microbiota and further investigated the potential of EO vapor in extending the shelf life of leafy green vegetables. Oregano EO vapor showed stronger antimicrobial activities than basil and clove EO vapors against common spoilage‐causing microorganisms in fresh produce, including *Pantoea agglomerans*, *Pseudomonas cichorii*, *Pectobacterium carotovorum*, *Pantoea ananatis*, *Pseudomonas marginalis*, *Alterneria bassicicola*, and *Botrytis cinerea*. When oregano EO vapor was applied to leafy greens, phytotoxic effects were observed on butter lettuce and iceberg lettuce but not on kai lan and kale. On the contrary, the green color and total chlorophylls of kai lan were preserved by oregano EO vapor. With the use of oregano EO vapor generated by 10 µL oregano EO in a 1.8‐L container, the shelf life of kai lan extended from 3 days to at least 7 days when stored at 25°C and from 5 days to at least 14 days when stored at 7°C. The antimicrobial effect was studied with the plating counting method to reflect the dose‐dependent amicrobial effect of oregano EO vapor on kai lan. High‐throughput 16S/18S DNA sequencing method was applied to evaluate the microbial ecological changes of kai lan under oregano EO vapor treatment. Taken together, our results suggest that oregano EO vapor could be used to extend the shelf life of kai lan. However, the dose of usage must be optimized with care, and one must realize the effectiveness might vary essentially when applying the EO vapor on other types of leafy green vegetables.

## 1 INTRODUCTION

Essential oils (EOs), fully described as plant‐based EOs, have been widely proposed as food preservatives due to their antimicrobial and antioxidative properties (Pandey et al., 2017). EOs comprise intricate blends of volatile compounds synthesized by plants with strong smells. These compounds can be found in various plant parts, including flowers, leaves, roots, fruits, seeds, wood, or bark (Pandey et al., 2017). For instance, the whole oregano grass has been extracted for aromatic oregano EO, while thyme EO is typically acquired from the leaves and flowers of *Thymus vulgaris* (Rajkovic et al., 2015). These EOs are characterized by the bioactive compounds, including terpenes, polyphenol, and flavonoid. These compounds are responsible for the antioxidant and antimicrobial activity of EOs. The application of EOs against food pathogens like *Staphylococcus aureus* and *Salmonella* spp. has been widely researched (Bajpai et al., 2012; Y. Zhang et al., 2016). However, less emphasis has been placed on the significance of microbial spoilage and quality deterioration on food under EO treatment.

The worldwide consumption of leafy greens can result in 135 billion USD revenue, with around 7.3% annual increasing rate (Batzios & Tsiouni, 2024). Microbial growth is a main cause of the spoilage of leafy greens. Leafy greens are highly affected by the microorganisms during harvest and post‐harvest period. Due to the direct contact of soil during growth, leafy greens generally contain high numbers of microorganisms, including both bacteria and fungi (Tournas, 2005). For instance, *Pseudomonas* spp., *Erwinia* spp., and *Acinetobacter* spp. have been identified as spoilage bacteria on leafy greens, resulting in soft rots, slime formation, and discoloration (Alegbeleye et al., 2022). Mold spoilage of leafy greens can be mainly caused by many species of *Penicillium*, *Alternaria*, *Botrytis*, and *Aspergillus*. Similarly, mold growth can also lead to the rotting and discoloration, while the growth of mold is more visible. Specifically, kai lan sampling in Vietnam has been detected with 9.28 log CFU/g of total aerobic plate count and 5.17 log CFU/g of total coliform (Minh, 2021). Kale (*Brassica oleracea* L. var. *acephala*), another leafy green that is highly similar to kai lan, is prone to browning and easily infected by aerobic bacteria, yeasts, and molds due to the minimal processing (Wang et al., 2022). In this case, it is important to apply preservation techniques to inhibit the microbial growth during the storage of leafy greens.

EOs have been widely proposed as food preservatives thanks to their antimicrobial activity, while their strong influence on food sensory qualities has limited their application especially when EOs were directly formulated in the foods or applied to the foods as liquids. Moreover, for leafy green vegetables, a water‐based preservative agent might introduce more moisture and encourage microbial deterioration on the contrary. Several in vitro studies have evaluated the antimicrobial activity of EO in both direct application and vapor application. For instance, *Citrus sinensis* EO has shown an MIC value of 800 mg/L (air) in vapor application and 1600 mg/L in liquid phase application against *Aspergillus flavus* (Velázquez‐Nuñez et al., 2013). *Eucalyptus* *globulus* EO has shown stronger antimicrobial activity in vapor application than direct application in both fungal and bacterial strains, with higher inhibition zone diameter under same concentration of EO treatment (Tyagi & Malik, 2011). EO has shown their strong potency in the vapor application for the antimicrobial activity against foods.

Therefore, this study aimed to investigate the preservative effects of EO vapor on leafy green vegetables. First, the antimicrobial activities of different types of EO vapor were evaluated against the selection of spoilage‐causing microorganisms from vegetables and fruits. Oregano EO vapor was then applied on several leafy greens to evaluate the appearance change under room temperature (25°C) and refrigeration (7°C) storage. The oregano EO vapor was further applied on kai lan to evaluate the shelf life extension effects evaluated from multiple perspectives.

## 2 MATERIALS AND METHODS

### 2.1 Chemical and biological materials

EOs extracted from oregano (*Origanum vulgare*), clove (*Eugenia caryophyllus*), and basil (*Ocimum basilicum*) were purchased from Now Foods in Singapore. Kai lan was purchased from a local urban farm (Vegeponics). Kale, butter lettuce, and iceberg lettuce were purchased from a local supermarket (Fairprice). Folin–Ciocalteau reagent, sodium carbonate, gallic acid, quercetin, and aluminum chloride were bought from Sigma‐Aldrich Chemical Company (Sigma‐Aldrich). Nutrient agar, nutrient broth, and potato dextrose agar were purchased from Oxoid. The pure cultures of five bacteria from American Type Culture Collection (ATCC), including *Pantoea agglomerans* (ATCC 27,155), *Pseudomonas cichorii* (ATCC 13,455), *Pectobacterium carotovorum* (ATCC 15,713), *Pantoea ananatis* (ATCC TSD‐232), *Pseudomonas marginalis* (ATCC 51,281), and two fungi (*Alterneria bassicicola* [ATCC 96,836] and *Botrytis cinerea* [ATCC 11,542]) were purchased from Everlife (Chemoscience Pte Ltd.). The selected EO was determined by gas chromatography (GC)–mass spectrometry (MS) analysis (Figure S1) following a protocol reported by Hai et al. (2022). The composition of oregano EO was determined by GC–MS (Agilent Scientific Instruments) with HP‐5MS 5% phenylmethyl siloxane capillary column (30 m × 0.25 mm × 0.25 µm). The initial temperature of the oven was set at 100°C, with increase rate of 5°C/min to 220°C, followed by 4°C/min to 250°C and 25°C/min to 300°C. At each temperature stage, the holding time was 5 min. The temperature for the injection inlet and detector was set at 250°C. The flow rate of 99% helium was set at 1 mL/min. The split ratio was 1:20. For the MS setting, the electron ionization mode was set at 70 eV, and the MS scan was from 35 m/z to 500 m/z.

### 2.2 Vapor‐phase antibacterial assay

The frozen bacteria cultures (−80°C) were activated by transferring them into 10 mL of nutrient broth and incubating for 24 h at relative temperatures as suggested by ATCC, respectively. An aliquot of the bacterial solution was inoculated onto a nutrient agar plate and incubated for 48 h to obtain several individual colonies, which were transferred into another 10 mL of nutrient broth for subculturing. All bacterial strains were subcultured for three times consecutively to prepare the ultimate bacterial inoculum for further experiments.

**FIGURE 1:**
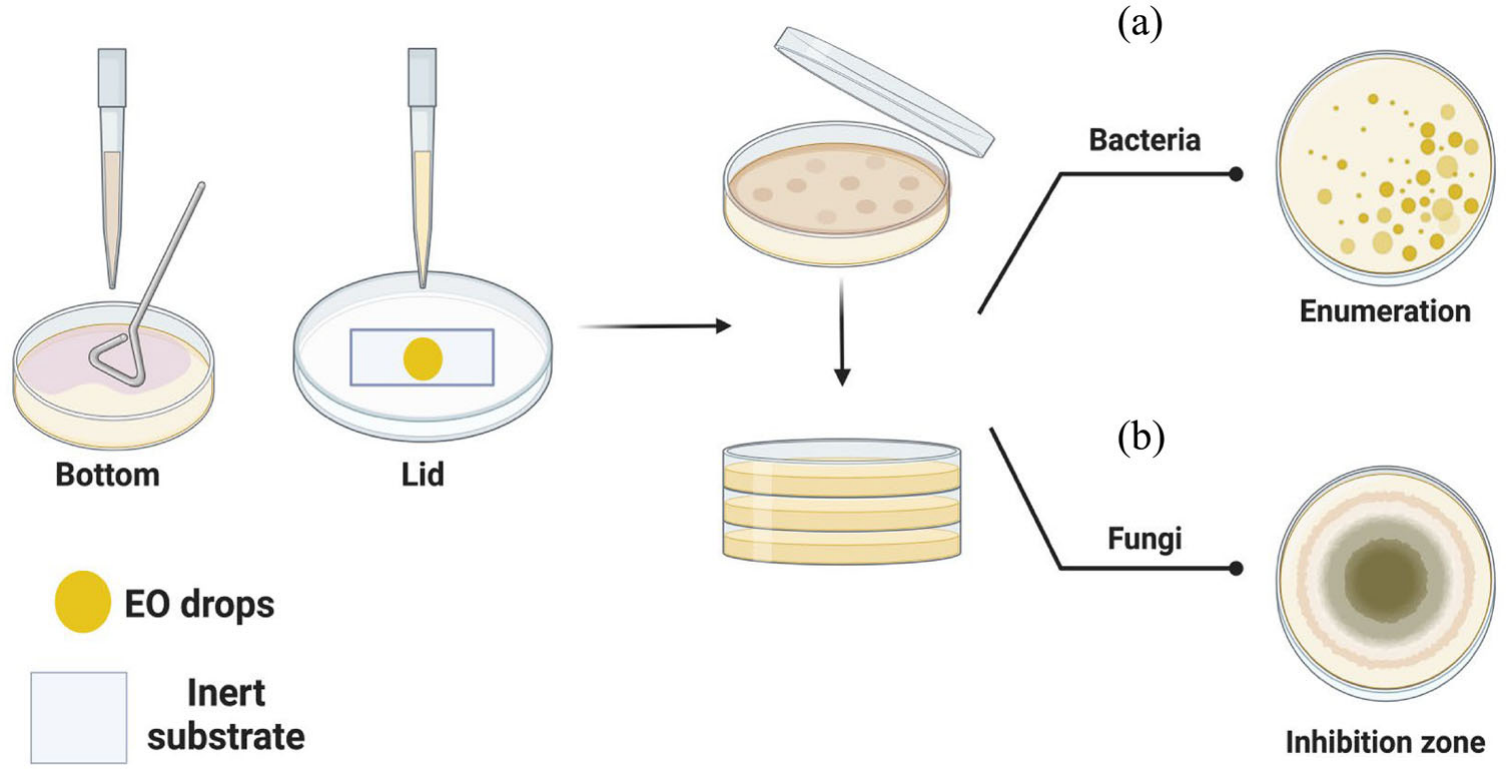
Schemes of vapor‐phase antibacterial assay (a) and antifungal assay (b) of essential oils (EOs) (created by BIORENDER).

Antibacterial tests of EO in vapor phase were established based on Mukurumbira et al. (2023) with slight modifications. For all the tested bacterial strains, the optical density at 600 nm (OD_600_) of each bacterial solution was measured by the BioDrop µLite Spectrophotometer to ensure the bacterial concentration was around 2–3 × 10^8^ CFU/mL as starting point. Solution was serially diluted 10^5^ times by 0.1% sterile peptone water, and 100 µL of the diluted solution was further inoculated on each nutrient agar. A glass cover slide was stuck onto the inside of Petri dish lid, and 2.5 µL of each EO was dropped and spread across the cover slide surface, respectively. Then, each agar plate set was sealed with parafilm and incubated under relative temperature for 48 h following the ATCC recommendations. Afterward, bacterial plate counts were enumerated, and the antimicrobial potencies of each EO vapor were indicated by their bacterial reduction ratio in contrast to the negative control (no EO applied). All the samples were tested in triplicate. The experimental setup is illustrated in Figure 1.

### 2.3 Vapor‐phase antifungal assays

For molds, the pure cultures were activated by plating on potato dextrose agar and incubated at 25°C for 5–7 days until the whole agar plate was visibly covered by the molds. The molds were subcultured for three cycles consecutively before the following experiments. The fungi cells were harvested by scraping off the agar surface with 10 mL of sterile 0.1% Triton X‐100 solution, obtaining the fungal suspensions for further work.

The mold suspension was directly inoculated on Dichloran‐rose Bengal Chloramphenicol agar. A glass cover slide was stuck on the lid of Petri dish, and a paper disk (diameter: 6 mm) was fixed at the lid center. EO was previously serially diluted by two times with dimethyl sulfoxide (DMSO), and 10 µL of each EO solution was dropped onto the paper disk, respectively. The lowest EO concentration that can induce a visible inhibition zone was recorded, and the size of inhibition zones was measured. Pure DMSO solution was used as the negative control and all tests were conducted in triplicate. The experimental setup is illustrated in Figure 1.

### 2.4 Application of EO vapor on leafy green vegetables

An inert plastic piece (4 × 8 cm) was adhered to the inner side of a commercial packaging container lid (volume: 1.8 L), on top of which a filter paper (diameter: 9 cm) was affixed to assist the EO vaporization. Fresh leaves (10 ± 0.5 g) of kai lan, kale, butter lettuce, or iceberg lettuce were placed at the bottom of each container, with their surfaces fully exposed to the EO vapor during storage. After that, EO was deposited onto the paper disk and the plastic containers were closed immediately. The sample containers were kept in the darkness at 25°C and 7°C, respectively, and the vegetables were sampled and analyzed at different time points.

### 2.5 Color measurement

The International Comission on Illumination (abbreviated CIE) L*a*b* coordinates were measured, and the indices, including color index (CI), total color difference (TCD), and yellowing index (YI) (Francis & Clydesdale, 1975; Goni et al., 2010), were calculated to properly characterize the evolution of vegetable surface color. Specifically, the a* values range from red (positive, +a*) to green (negative, −a*), the b* values vary from yellow (positive, +b*) to blue (negative, −b*), and the L* values (lightness) are from black (*L* = 0) to white (*L* = 100). Prior to the measurements, the instrument was calibrated using a standard white reference plate (L* = 41.103, a* = −4.743, and b* = 4.800). Each leaf was measured at four similar positions, and triplicates of each type were examined (total *n* = 12) using a Minolta CR‐100 Colorimeter Reflectance Spectrophotometer with a D65 illumination source (Minolta Camera Co.). Each spot was measured three times, and the automatic average values were reported. The color indices were calculated as follows (1–3):

(1) $$\mathrm{CI}=1000\times a^{*}/L^{*}/b^{*}$$

(2) $$\mathrm{TCD}=\sqrt{{\left(L^{*}-L_{0}^{*}\right)}^{2}+{\left(a^{*}-a_{0}^{*}\right)}^{2}+{\left(b^{*}-b_{0}^{*}\right)}^{2}}$$

where L*, a*, and b* were values of differently treated kai lan, and L_0_, a_0_, and b_0_ were the values of fresh kai lan without EO fumigation (the control) on Day 0 (Manolopoulou & Varzakas, 2016).

(3) $$\mathrm{YI}=142.86\times b^{*}/L^{*}$$

where YIs were calculated based on the b* values (yellow–blue axis) and L* (lightness) values, respectively.

### 2.6 Total chlorophyll content measurement

Chlorophylls are principal plant pigments that predominantly influence the visual green color of kai lan leaves, and their contents were determined based on a previous protocol reported elsewhere (Huang et al., 2021). Kai lan leaves after oregano EO treatment and storage were lyophilized, followed by grinding into powder. Around 10 ± 0.1 mg of freeze‐dried kai lan powder was dissolved in 10 mL of aqueous acetone solution (acetone:water = 80:20, v/v) and ultrasonicated for 15 min (by Elmasonic S 60H). Final solution was stored under −20°C for 24 h, respectively. The mixtures were further centrifuged at 3500 × *g* for 10 min at 4°C, and the absorbances of the supernatant were measured at 663.6 (A_663.6_), 646.6 (A_646.6_), and 440.5 nm (A_440.5_) using a the BioDrop µLite Spectrophotometer (Biodrop). The concentrations of chlorophylls (Chl) were quantified in milligrams per gram of dry weight (DW) based on the following formulas (4–6):

(4) $$\mathrm{Chl}\;a\;\mathrm{content}\;=\frac{12.25A_{663.6}-2.55A_{646.6}}{\mathrm{DW}}$$

(5) $$\mathrm{Chl}\;b\;\mathrm{content}\;=\frac{20.31A_{646.6}-4.91A_{663.6}}{\mathrm{DW}}$$

(6) TotalChlcontent=Chlacontent+Chlbcontent

### 2.7 Total microbial aerobic count measurement

For the total aerobic counts (TAC) tests, 10 g of treated vegetables were transferred to a sterile stomacher bag (Delta lab, 180 × 300 mm), and thoroughly mixed with 90 mL 0.1% peptone water by stomacher (Masticator Stomacher, IUL Instruments). Then, the mixture was 10 times serially diluted, and the same extract was inoculated on both the plate count agar (for TAC) and then incubated at 30°C for 2 days. TAC results were expressed in log CFU/g vegetables, and samples were analyzed in triplicate. The rest of the mixture for the kai lan leaves and peptone water was transferred to a 50‐mL falcon tube for further DNA extraction and sequencing in the next section.

### 2.8 DNA extraction and 16S/18S sequencing

DNA extraction was conducted based on Dakwa et al. (2021) with slight modifications. The mixture in 50‐mL falcon tubes was first centrifuged at 1500 × g for 10 min, and the supernatant was extracted and further centrifuged (Greiner Bio‐one) for 35 min at 3900 × *g* to collect the bacterial cell pellets. All the supernatant was discarded, and the sediment pellet was washed two times using 5 mL 1% PBS solution followed by centrifugation at 8000 × g for 5 min. Supernatant was further removed, and the cell pellet was resuspended in 1 mL PBS. This bacterial suspension was transferred to a 1.5‐mL microcentrifuge tube and then stored at −80°C for the following (approximately 2 weeks) DNA extraction.

DNA was extracted using the DNeasy PowerFood Microbial Kit (Qiagen Singapore Pte. Ltd.) according to the manufacturer's protocol. Bacteria cells in PBS solution were thawed at room temperature (RT, 25°C) and transferred to the collection tube provided by the kit. Cells were centrifuged at 13,000 × *g* for 1 min, and the supernatant was decanted using a pipette tip. Microbial DNA was extracted according to the protocol. The concentration and purity of extracted DNA were assessed by the BioDrop µLite Spectrophotometer. Extracted DNA was contained in a 1.5‐mL microcentrifuge tube and stored at ‐80°C before sending for the sequencing analysis.

Extracted DNA was sent to NovogeneAIT Genomics Singapore Pte Ltd. for 16S V3‐V4 amplicon and 18S V4 amplicon sequencing via the Illumina NovaSeq 6000. Quality control, polymerase chain reactions (PCR), library preparation, and bioinformatics analysis pipeline were conducted accordingly. Data were analyzed via the open‐source Quantitative Insights into Microbial Ecology 2 pipeline for the denoise to obtain initial amplicon sequence variants and species annotation via the Silva Database (Bokulich et al., 2013; Li et al., 2022). The top 10 taxa of each sample or group at each taxonomic rank (phylum, class, order, family, genus, and species) were used to plot the distribution histogram of relative abundance in Perl through Scalable Vector Graphics (SVG) function, which visually displays different abundance and taxa clustering. Genus's rank of 16S amplicon sequencing and family's rank of 18S amplicon sequencing were presented in discussion. Non‐metric multidimensional scaling (NMDS) was conducted to comparatively analyze the differences in compositions of microbial community among tested groups through R software with the ade4 package (Rivas et al., 2013). To determine species with significant variations between groups, *t*‐test was performed at genus rank for 16S amplicon sequencing and family rank for 18S amplicon sequencing, respectively.

### 2.9 Statistical analysis

All the tests were performed for three times independently and were analyzed by one‐way analysis of variance and paired‐samples *t*‐test by IBM SPSS statistic 25.0. The significance of difference was *p <* 0.05.

## 3 RESULTS AND DISCUSSION

### 3.1 Antimicrobial effect of EO vapor on spoilage‐causing microorganisms

All the tested EO vapors have shown noticeable antibacterial activities against the tested bacteria as indicated by their plate count reduction (%) (Table 1). Among three tested EO, oregano EO showed stronger antibacterial effects than clove EO and basil EO against all five tested bacteria. The inhibition of EO vapors on the tested strains used in this work has been rarely explored so far. Nonetheless, the efficacies of oregano EO against *Pseudomonas syringae*, a vegetable spoilage bacterium, were reported previously (Carezzano et al., 2017). Oregano EO could inhibit the biofilm formation and phytotoxin (coronatine, syringomycin, and tabtoxin) production based on an in vitro study.

**TABLE 1:** Overall antibacterial and antifungal activities of oregano, clove, and basil essential oil (EO) vapors.

Pure cultures	Oregano		Clove		Basil	
Bacteria	Plate count reduction (%)	Ranking^a^	Plate count reduction (%)	Ranking^a^	Plate count reduction (%)	Ranking^a^
*Pantoea ananatis*	52.91 ± 10.28a	1	29.82 ± 13.88b	2	26.17 ± 15.45b	3
*Pantoea agglomerans*	80.24 ± 2.79b	2	62.40 ± 5.47d	3	91.91 ± 1.54a	1
*Pseudomonas cichorii*	71.14 ± 13.36ab	1	57.59 ± 17.81bc	2	49.92 ± 11.68c	3
*Pectobacterium carotovorum*	62.90 ± 5.64a	1	55.03 ± 3.40b	3	62.50 ± 4.25a	2
*Pseudomonas marginalis*	62.02 ± 12.43ab	2	70.45 ± 12.01a	1	53.83 ± 17.57b	3
**Fungi**	**Lowest effective volume (µL)**	**Ranking**	**Lowest effective volume (µL)**	**Ranking**	**Lowest effective volume (µL)**	**Ranking**
*Botrytis cinerea*	1.25	1	1.25	1	20.00	2
*Alterneria bassicicola*	2.50	2	1.25	1	>20.00	3
Total ranking^b^	10		13		17	

^a^ Ranking 1 indicates the most antimicrobial EO, followed by the moderate (score of “2”) and the least (score of “3”) antimicrobial EO.

^b^ The lower ranking score indicates the higher overall antimicrobial activities.

Antibacterial mechanisms of EOs have been widely investigated and normally involve several aspects. Major components of EOs are terpenes, alcohols, esters, and phenolic compounds, which can interact with cell membrane components and disrupt the cell membrane integrity (Pandey et al., 2017). Oregano EO can increase the cell membrane electrical conductivity and reduce intracellular protein concentrations of *S. aureus*, which reflects the cell membrane disruption and leakage of cellular components (Cui et al., 2019). EO can also interact with DNA components and affect the gene expression in bacterial cells. EO vapor has similar antibacterial mechanisms, where they exert greater antibacterial efficacies than EOs in the liquid form (Reyes‐Jurado et al., 2020). This may be because the lipophilic EO forms micelles in an aqueous phase, which restrains the interactions between active EOs and bacteria (Nadjib et al., 2014). Additionally, EO in the vapor phase can mitigate their interaction with food matrices, which alleviates the unwanted changes in sensory and odor attributes of foods during EO preservation.

Two notorious fungal pathogens, *A. bassicicola* and *B. cinerea*, can plague a wide range of fruits and vegetables (Leifert et al., 1993; Soylu et al., 2010). In this work, the three EOs were serially diluted and their antifungal effects in the vapor phase were evaluated based on the lowest effective volume within each Petri dish (Table 1), and the dimensions of relative inhibition zone (ZOI) (Figure S2). EO was deposited at the center of each Petri dish lid and a circle of inhibition zone appears when the EO vapors can effectively inhibit the fungal growth. According to Table 1, the oregano and clove EO vapors exhibited similar antifungal activities as indicated by their proximate lowest effective volume (1.25–2.5 µL) that can cause similar ZOI. In general, *A. bassicicola* showed stronger resistance compared to *B. cinerea* against the oregano and basil EO vapors.

Antifungal mechanisms of EOs are similar to those against bacteria, while the different fungal cell structures may render them higher resistance against EO (Sekyere & Asante, 2018). General antifungal mechanisms of EOs involve cell membrane disruption (Gogoi et al., 1997), mitochondria dysfunction (Chen et al., 2013), and reactive oxygen species production (Nazzaro et al., 2017). According to the literature, low levels of oregano EO both in the vapor (0.2 µg/mL air) and in liquid contact phase (12.8 µg/mL liquid) can effectively inhibit *B. cinerea* (Soylu et al., 2010). The antifungal activities of EOs were attributed to their possible accumulation in the lipophilic components within fungal cell membranes (Nazzaro et al., 2017). This accumulation facilitates the subsequent translocation of other EO components to the intracellular milieu. The variations in antifungal efficacies between different EOs result from their diverse physicochemical properties, especially their water solubility and lipophilicity (X. M. Xie et al., 2004). Interestingly, clove EO, when tested in the liquid phase against *A. bassicicola*, resulted in the abnormal growth of mycelia and swollen hypha (Peddi et al., 2022; Suwitchayanon & Kunasakdakul, 2009). By contrast, the antifungal activities of clove EO vapors were noticeable in this work, highlighting the advantages of EO vapors as antimicrobial agents.

To compare the overall antimicrobial activities of different EOs and further select the EO applied for shelf life extension tests on kai lan, the antimicrobial activities of the EO vapors were scored and ranked (Table 1). The EO with strongest antimicrobial activity against the strain among tested EOs was scored as 1, followed by 2 and 3. The total scores for all the EO were calculated as the sum of scores for five bacterial strains and three fungal strains. According to Table 1, oregano EO has the lowest total score of 10 followed by 13 for clove EO and 17 for basil EO. Therefore, oregano EO with the strongest antimicrobial activity was selected for further screening and experiments.

The chemical composition of oregano EO used in this work was analyzed by GC–MS. As shown in Figure S1, the main component of oregano EO was carvacrol, accounting for 85.78% of total content, followed by 7.19% of cymene and 2.56% of linalool. This composition was comparable to other finding, showing that the major component of oregano EO was carvacrol (63.97%), p‐cymene (12.63%), and linalool (3.67%) (Özkan et al., 2017).

### 3.2 Application of oregano EO vapor on leafy vegetables

Four leafy greens commonly consumed in Singapore (kai lan, kale, butter lettuce, and iceberg lettuce) were chosen to examine the applicability of the oregano EO vapor for their preservation. To identify the applicable EO dose and suitable vegetables, 10 or 75 µL of oregano EO was added into each container (1.8 L), and the vegetables were stored at 7°C for 5 days. As shown in Figure S3A, the green color of kai lan leaves can be preserved using oregano EO vapor at a low dose of 10 µL EO in 1.8 L, which effectively delayed its yellowing in contrast to the control (no EO). However, a high dose of oregano EO vapor (75 µL EO in 1.8 L) caused noticeable darkening of leaves. This was possibly attributed to the accumulation of phenolic compounds (Kraśniewska et al., 2016; Saltveit et al., 2005). Figure S3B shows that the kale leaves exhibited a color‐change pattern similar to those of kai lan leaves (Chinese kale), as they are both from the *Brassicaceae* family. However, the green color preservative effect on kale was not as apparent as that on kai lan. In contrast, oregano EO vapor did not provide any noteworthy benefits in preserving the appearance of butter lettuce and iceberg lettuce (Figure S3C,D). Oregano EO vapor has shown phytotoxic effects toward these two kinds of lettuce, even at the low dosage (10 µL EO in 1.8 L) treatment. Such phytotoxic effects of oregano EO have been observed against the crop seeds of radish, wild mustard, and also the weed of Italian ryegrass, especially under high concentration treatment (1 and 0.5 g/mL) (Amato et al., 2023). Potential reasons for the phytotoxic effects of oregano EO are the inhibition of α‐amylase activity and the interaction with plant cell membrane under high dosage (Amato et al., 2023). Additionally, the seed germination of cucumber and tomato was inhibited by oregano EO under 0.5 µL/mL concentration treatment. Thus, with consideration of both green color preservative and phytotoxic effects of oregano EO, kai lan was chosen as the target matrix for further studies. As for the dosage, 10 µL EO in 1.8 L was selected as it produced notable color preservative effects, and 75 µL EO in 1.8 L was not tested further as it resulted in grayish kai lan leaves. Instead, a medium dosage of 40 µL EO in 1.8 L was tested.

### 3.3 Surface color determination of kai lan leaves

**FIGURE 2:**
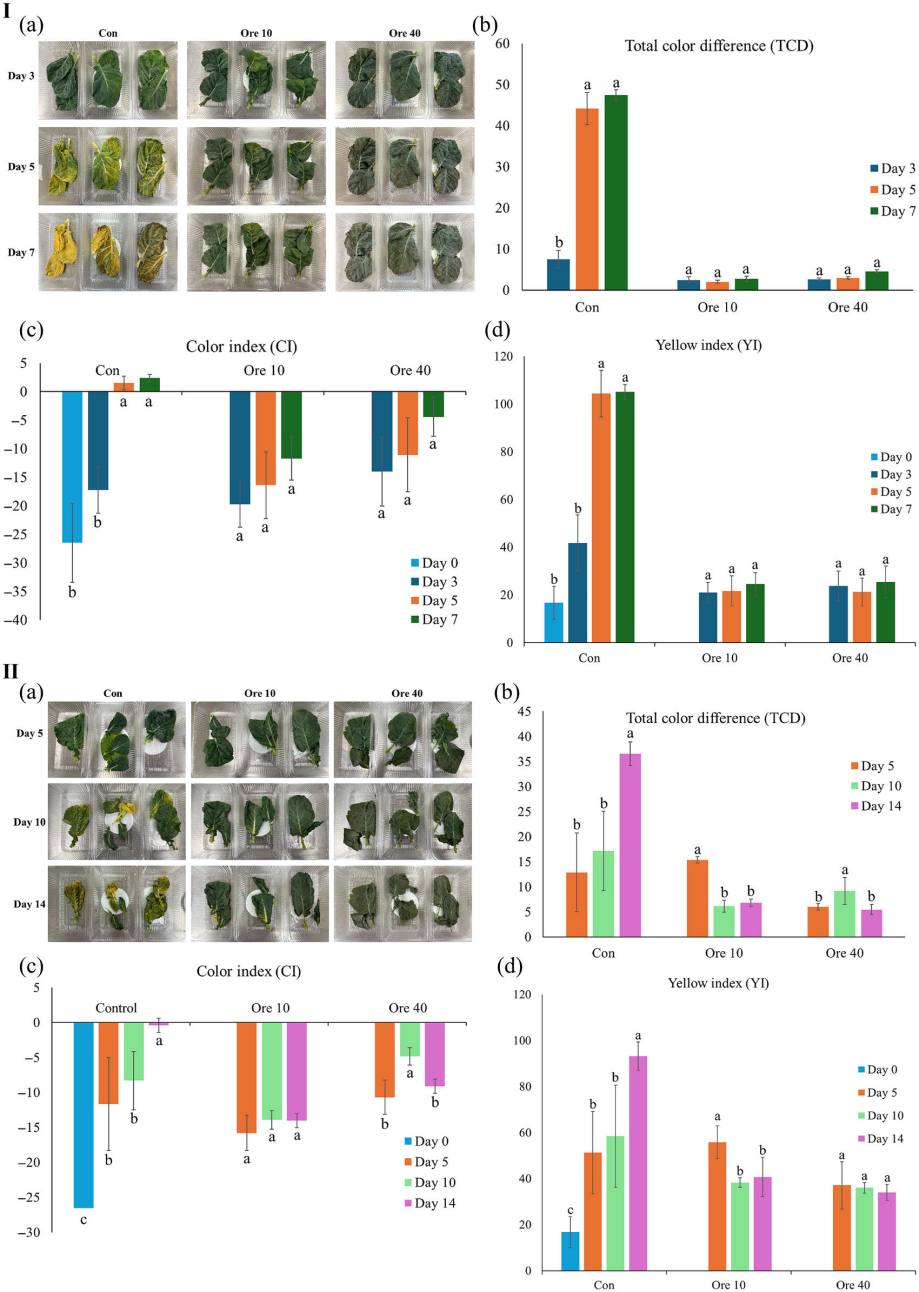
Surface color determination of kai lan under oregano essential oil (EO) treatment stored at 25°C (I) and 7°C (II). (a) Photos of kai lan leaves after storage; (b) total color difference; (c) color index; and (d) yellow index. Con: control without EO; Ore 10: 10 µL oregano EO in 1.8 L; Ore 40: 40 µL oregano EO in 1.8 L. Means ± standard deviations (*n* = 3) are used to present the data. The means with different letters under certain treatment indicate a significant difference (*p *< 0.05).

Deterioration in the visual quality of leafy greens typically manifests as the unfavorable color changes such as yellowing. Both surface color and total chlorophyll content have been widely determined to characterize the color changes of leafy vegetables (Barrett et al., 2010). To characterize the color and its changes during storage, CI, TCD, and YI were determined. CI mainly focuses on the absolute color value of an object, indicating the real color of the sample (López Camelo & Gómez, 2004). TCD is a numerical description of the color difference between two colors, while YI mainly describes the yellowness of the samples, which can provide more direct information about the yellowness of kai lan leaves during the storage (Jung & Sato, 2013; Wu et al., 2021). The CI of kai lan under oregano EO vapor treatment is shown in Figure 2I(c). According to the standard evaluation, CI (−40 to −20) represents blue violet to dark green, CI (−20 to −2) indicates dark green to yellowish green, CI (+2–+20) shows pale yellow to deep orange, and CI (+20 to +40) stands for deep orange to deep red (GoÑI et al., 2010). After the 7‐day storage at 25°C, the CI value of kai lan in the control group (no EO) markedly increased from −26.50 ± 6.89 to 2.36 ± 0.65 (*p* < 0.05), indicating that its color changed from dark green to pale yellow. By contrast, in the presence of oregano EO vapors, the color change of kai lan was significantly retarded as indicated by the slower increases in their CI values. This was consistent with TCD (Figure 2I[b]) and YI (Figure 2I[d]), both of which significantly decreased under the EO vapor treatment. Particularly, the oregano EO vapor showed the lowest TCD and YI values, being the most effective to preserve the initial green color of kai lan against the following color changes and yellowing. These results were consistent with visual observations, confirming that the oregano EO vapor is promising to preserve the green color of kai lan.

It has been shown that refrigeration storage can effectively retard the yellowing progress of several leafy greens such as lettuce, broccoli (Manolopoulou & Varzakas, 2016), baby spinach (Y. Kou et al., 2014; Y. Xie et al., 2021), and rocket leaves (Kim & Ishii, 2007). Therefore, kai lan storage with oregano EO vapor was also tested at 7°C for 14 days. Similar color protection effects of oregano EO vapor were observed (Figure 2II). The TCD was in the range of 5–15 under oregano EO vapor treatment and this value was about 36 for the control group (compared to the Day 0 value; Figure 2II[b]). Based on Figure 2II(c,d), the yellowish effects of kai lan leaves were remarkably inhibited by oregano EO vapor treatment.

### 3.4 Total chlorophyll content of kai lan

**FIGURE 3:**
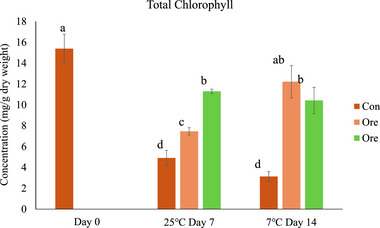
Total chlorophyll content (mg/g dry weight [DW]) of kai lan under oregano essential oil (EO) vapor treatment after storage at 25°C for 7 days and 7°C for 14 days. Con: control without EO; Ore 10: 10 µL oregano EO in 1.8 L; Ore 40: 40 µL oregano EO in 1.8 L. Means ± standard deviations (*n* = 3) are used to present the data. The means with different letters indicate a significant difference (*p* < 0.05).

The overall discoloration of leafy greens is preliminarily associated with the losses of chlorophylls, which simultaneously magnifies the appearance of leaf yellowing during storage. The decreasing chlorophyll content was consistent with the fading of green color (Figure 3). For the control group (no EO), the total chlorophylls in kai lan decreased from 15.39 ± 1.38 mg/g DW to 4.91 ± 1.60 mg/g DW (reduced by 68.10%; *p* < 0.05) over 5 days at 25°C and reached a plateau till Day 7. With a dose of 10 µL oregano EO in 1.8 L, the total chlorophylls in kai lan were markedly maintained with no significant reduction after 14 days of storage at 7°C (*p* > 0.05). After the 7 days of storage at 25°C, the dose of 40 µL EO in 1.8 L provided better protection against the chlorophyll decreases in kai lan than 10 µL EO in 1.8 L EO vapor (*p* < 0.05). The maintenance of chlorophyll content in kai lan leaves of treatment groups can be due to the antioxidative effects of oregano EO and the inhibition of oxygen permeation with oregano EO vapor surrounding the kai lan leaves (Abedi et al., 2021).

### 3.5 The microbial analysis of kai lan under oregano EO vapor treatment

Apart from the favorable color preservation, it was indeed essential to investigate the effects of oregano EO vapors on the microbial quality of kai lan regarding their ultimate shelf life extension in real‐life scenarios.

**FIGURE 4:**
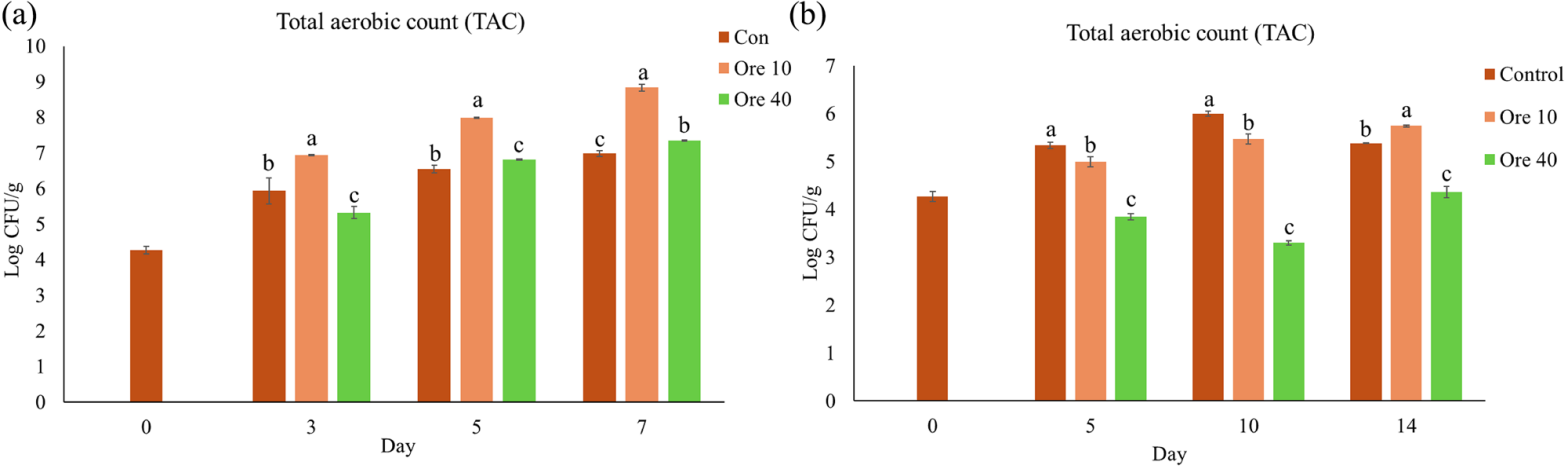
Total aerobic counts (TAC) of kai lan exposed to oregano essential oil (EO) vapor stored at 25°C (a) and at 7°C (b). Con: control without EO; Ore 10: 10 µL oregano EO in 1.8 L; Ore 40: 40 µL oregano EO in 1.8 L. Means ± standard deviations (*n* = 3) are used to present the data. The means with different letters at certain time point indicate a significant difference (*p* < 0.05).

Although oregano EO vapor showed significant inhibition to all the five tested spoilage‐causing bacteria strains and two fungal pathogens, as shown in Section 3.1, the antimicrobial effect was not fully demonstrated by measuring the TAC on kai lan with complicated natural microbiome. As shown in Figure 4, significantly lower microbial loads in oregano EO vapor‐treated groups than in the control group were only noticed on Day 3 at 25°C with the dose of 40 µL oregano EO in 1.8 L (Figure 4a; *p* < 0.05), on Days 5 and 10 at 7°C with the dose of 10 µL oregano EO in 1.8 L, and on Days 5, 10, and 14 at 7°C with the dose of 40 µL oregano EO in 1.8 L (Figure 4b; *p* < 0.05). We hypothesized this was largely due to the various susceptibilities of different microbes on kai lan, and thus it became necessary to investigate the microbial composition changes before and after oregano EO vapor treatment.

**FIGURE 5:**
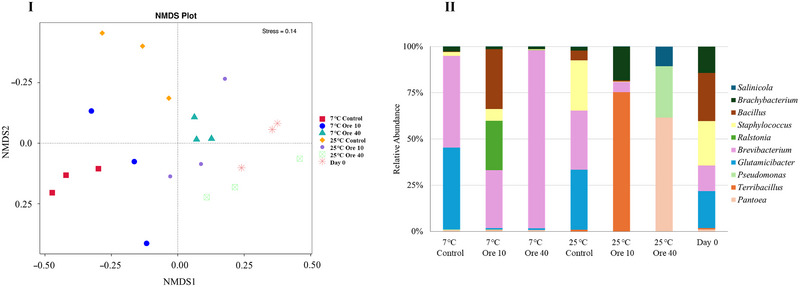
Bacterial biota of kai lan with and without oregano essential oil (EO) vapor before and after the storage at 25°C for 7 days or at 7°C for 14 days. (I) Non‐metric multidimensional scaling (NMDS) based on unweighted unifrac distance of 16S amplicon sequencing results; (II) relative abundance in bacteria genus. Con: control without EO; Ore 10: 10 µL oregano EO in 1.8 L; Ore 40: 40 µL oregano EO in 1.8 L.

The NMDS analysis of 16S amplicon sequencing results from different groups (doses of EO vapor and storage temperatures) is shown in Figure 5I. Stress smaller than 0.2 indicates that this scaling result is reliable. It was interesting to find that the samples treated with oregano EO vapor with the dose of 40 µL oregano EO in 1.8 L at both 25°C and 7°C showed more similar patterns to the samples on Day 0 than other groups. The samples treated with lower dose of EO vapor (10 µL oregano EO in 1.8 L) showed more similar patterns to the control groups without EO vapor treatment than the samples treated with higher dose of EO vapor (40 µL oregano EO in 1.8 L) at 25°C and 7°C, respectively. However, the relative abundance in bacteria genus as shown in Figure 5II still showed the distinctive microbial compositions in samples treated with different doses of EO vapor and different storage temperatures. In comparison with the major genus identified on Day 0, *Terribacillus* became more competitive after being stored at 25°C for 7 days with the dose of 10 µL oregano EO in 1.8 L. *Pantoea*, *Pseudomonas*, and *Salinicola* dominated the bacterial components in kai lan stored at 25°C with the dose of 40 µL oregano EO in 1.8 L. After the kai lan was stored at 7°C for 14 days, *Ralstonia* became more competitive with the dose of 10 µL oregano EO in 1.8 L, whereas with the dose of 40 µL oregano EO in 1.8 L, *Brevibacterium* remained the dominant genus in the bacteria on kai lan after the storage. Taken together, these results indicated indeed a dose‐dependency in the antibacterial effects of oregano EO vapor when used to treat kai lan with natural microbiome.

**FIGURE 6:**
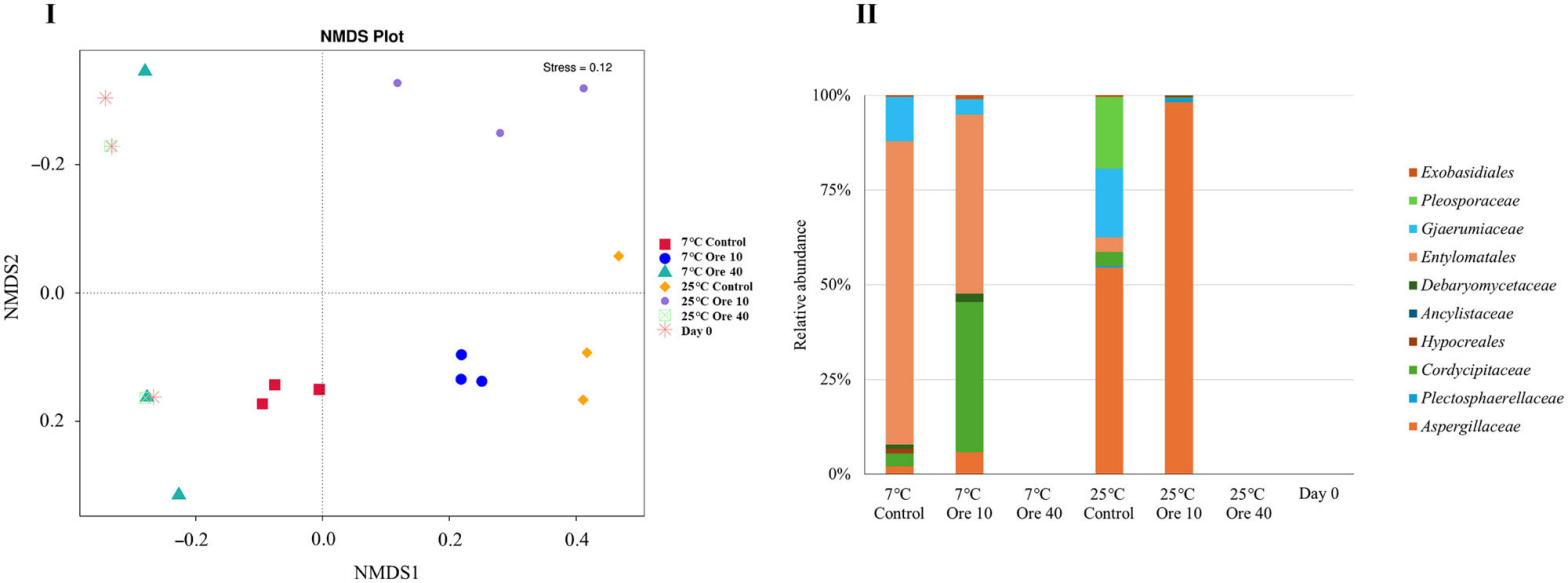
Fungal biota of kai lan with and without oregano essential oil (EO) vapor before and after the storage at 25°C for 7 days or at 7°C for 14 days. (I) Non‐metric multidimensional scaling (NMDS) based on unweighted unifrac distance of 18S amplicon sequencing results; (II) relative abundance in fungi family. Con: control without EO; Ore 10: 10 µL oregano EO in 1.8 L; Ore 40: 40 µL oregano EO in 1.8 L.

The NMDS analysis of 18S amplicon sequencing results from different groups (doses of EO vapor and storage temperatures) is shown in Figure 6I. Stress smaller than 0.2 indicates that this scaling result is reliable. The samples treated with oregano EO vapor with the dose of 40 µL oregano EO in 1.8 L at both 25°C and 7°C showed more similar patterns to the samples on Day 0 than other groups. As shown in Figure 6II, no fungi were identified from the samples on Day 0 and samples treated with oregano EO vapor with the dose of 40 µL oregano EO in 1.8 L at both 25°C and 7°C, whereas various fungi families were identified from the control groups without EO treatment stored at 25°C for 7 days and 7°C for 14 days, as well as the samples treated with oregano EO vapor with the dose of 10 µL oregano EO in 1.8 L. After being stored at 25°C for 7 days, *Aspergillaceae, Pleosporaceae*, and *Gjaerumiaceae* were found to be abundant on kai lan without EO treatment and with oregano EO vapor with the dose of 10 µL oregano EO in 1.8 L. When the kai lan was stored at 7°C for 14 days, *Entylomatales* and *Cordycipitaceae* were more enriched in the samples without EO treatment and with oregano EO vapor with the dose of 10 µL oregano EO in 1.8 L. Similarly to the bacterial sequencing analysis, these results indicated a dose‐dependency in the antifungal effects of oregano EO vapor when used to treat kai lan with natural microbiome.

Limited research discussed the application of oregano EO vapor on leafy greens, while the antimicrobial and phytotoxicity effects of oregano EO have been studied. Oregano EO vapor has presented stronger antimicrobial activity against Gram‐negative bacteria compared to cinnamon EO vapor and thyme EO vapor, reflected from lower MIC value against same bacteria strain (López et al., 2007). In the actual application, oregano EO combined with rosemary EO has been applied to iceberg lettuce and chard in liquid form and resulted in significant log reductions of food pathogens related to leafy greens, including *Listeria monocytogenes*, *Escherichia coli*, and *Salmonella* *enteritidis* (de Medeiros Barbosa et al., 2016). The seed germination of cucumber and tomato under EO treatment has been evaluated and oregano EO has shown strongest inhibitory effects of tomato seed germination among tested EOs, with 0.125 µL/mL treatment leading to 50% reduction of seed germination (Ibáñez & Blázquez, 2020). This indicates that oregano EO has strong phytotoxic effects against tomatoes. In this case, oregano EO vapor can be applied as antimicrobials against kai lan, while the dosage needs to be properly controlled to avoid the negative phytotoxic effects of oregano EO.

## 4 CONCLUSION

In order to evaluate the potential of EO vapor in extending the shelf life of leafy green vegetables, the antimicrobial effects of oregano, clove, and basil EO vapors were first tested against five bacterial (*P. agglomerans*, *P. cichorii*, *P. carotovorum*, *P. ananatis*, *P. marginalis*) and two fungal (*A. bassicicola* and *B. cinerea*) strains that have been previously recognized as spoilage‐causing microorganisms of fresh produce. Oregano EO vapor was selected to proceed with the application on the vegetables as it showed the highest antimicrobial effect overall on the pure culture. Within the four leafy green vegetables tested (kai lan, kale, butter lettuce, and iceberg lettuce), kai lan suffered the least from the phytotoxic effect, whereas in the meantime, it benefited largely from the oregano EO vapor treatment, which slowed down its losses of chlorophylls and thus maintained its favorable green color. These color protective effects were further demonstrated with different storage temperatures at 25°C and 7°C, and with different dose of EO vapor generated from 10 and 40 µL of oregano EO in 1.8‐L containers. The antimicrobial effect was studied with both the plating counting method which enumerates the cultivable microorganisms on kai lan and high‐throughput sequencing method. As a result, the culture‐independent method was able to better reflect the dose‐dependent amicrobial effect of oregano EO vapor on kai lan.

Important lessons were learned in this study, particularly from applying the EO vapor in genuine food samples. Although the antimicrobial property of EO is widely used to justify the use of EO as food preservatives, one must never forget the possible side effects of the antimicrobial agents on the food quality. In this study, strong phytotoxic effect was observed on butter lettuce and iceberg lettuce, suggesting the unsuitability of using EO vapor on these types of vegetables. On the other hand, a large number of studies in the literature spike pure culture of spoilage‐causing microorganisms on sterile food models to evaluate the antimicrobial effect of EO (Hai et al., 2022; Lou et al., 2024; C. Zhang et al., 2023). Although being more robust and repeatable, these studies excluded the implicit factors from the natural microflora on the foods. In this study, our results demonstrated the essential gaps between the effect on pure culture and on the natural microflora on foods, justifying the necessity of maintaining the natural flora on foods for future experimental setups of such studies. For the further experiments and up‐scale industry, the proper dosage of oregano EO vapor for post‐harvest preservation of kai lan is in the range of 10–40 µL/1.8 L of air applying to 10 g of kai lan leaves. It is also critical to conduct sensory evaluation to evaluate the consumer acceptance of kai lan after oregano EO vapor treatment. Additionally, we should also consider combining other packaging techniques like nano‐packaging with oregano EO vapor for effective release of EO vapor and better preservative effects against kai lan.

## AUTHOR CONTRIBUTIONS

**Weichen Shu**: Conceptualization; methodology; data curation; investigation; writing—original draft. **Zhuoliang Deng**: Data curation; investigation. **Lingdai Liu**: Conceptualization; methodology; investigation; validation; writing—original draft. **Jiaxuan Zhang**: Investigation; methodology. **Dan Li**: Conceptualization; validation; project administration; funding acquisition; writing—review and editing; resources; supervision.

## CONFLICT OF INTEREST STATEMENT

The authors declare no conflicts of interest.

## Supporting information



Supporting Information

## Data Availability

Data will be made available on request.
