# Supplementary material for: Effectiveness of oregano essential oil vapor on shelf life extension of kai lan (Brassica oleracea var. alboglabra)
Source: J Food Sci. 2025 Jan 19;90(1):e17673. doi: 10.1111/1750-3841.17673 (PMC11743022; doi:10.1111/1750-3841.17673)
Supplement: Supplementary file 1 — Supporting Information [file JFDS-90-0-s001.docx]

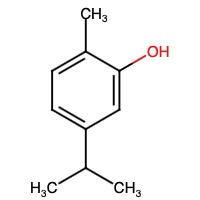


**Figure S1.** GC-MS profile of oregano EO composition.

**Table 1.** Composition of oregano EO determined by GC-MS.

| Retention time (min) | Compound | Area (%) |
| --- | --- | --- |
| 14.046 | cymene | 7.19 |
| 14.789 | terpinene | 2.39 |
| 15.545 | linalool | 2.56 |
| 21.482 | carvacrol | **85.78** |
| 26.080 | caryophyllene | 2.08 |

**
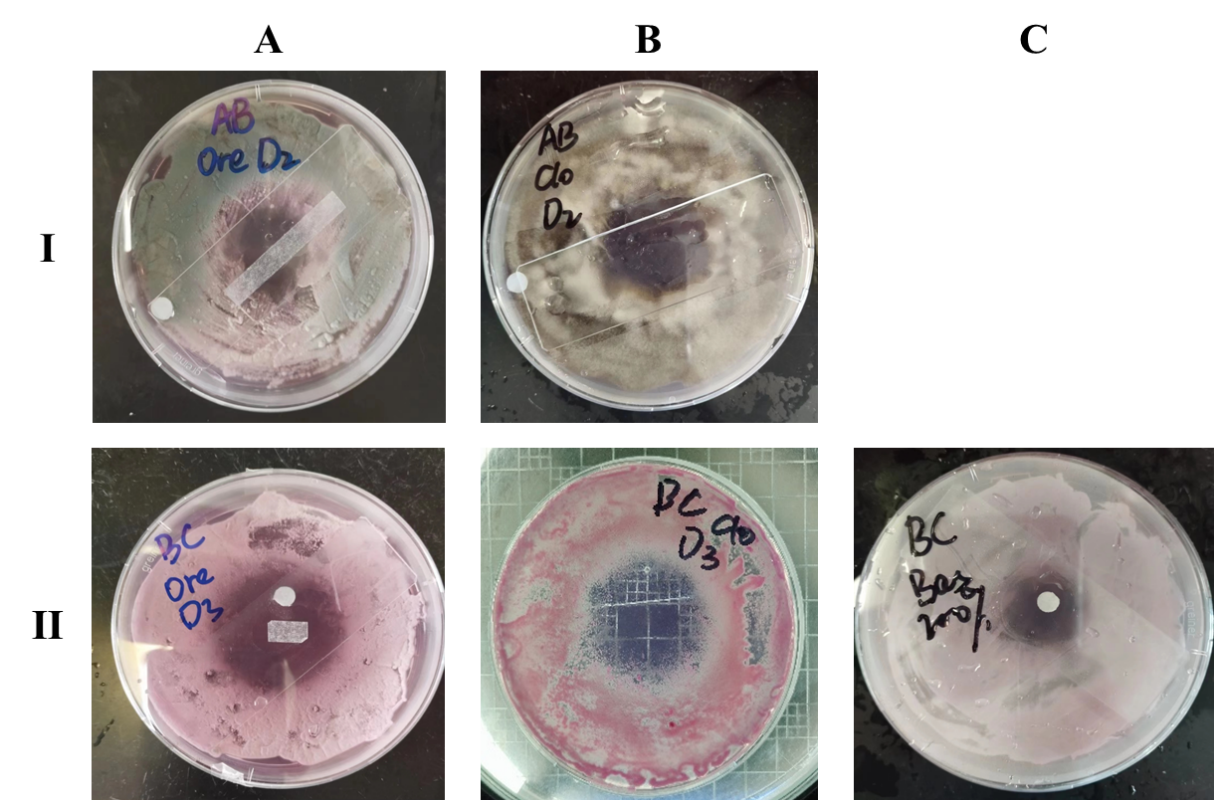
**

**Figure S2.** Formation of zone of inhibition (ZOI) in the presence of EO vapors against (I) *Alterneria bassicicola* and (A: 2.5 µL oregano EO; B: 2.5 µL clove EO) (II) *Botrytis cinerea* at lowest effective volume (A: 1.25 µL oregano EO; B: 1.25 µL clove EO; C: 20 µL basil EO). Notes: Basil EO did not show any anti-fungal activity against *Botrytis cinerea* with all the treatment volume.


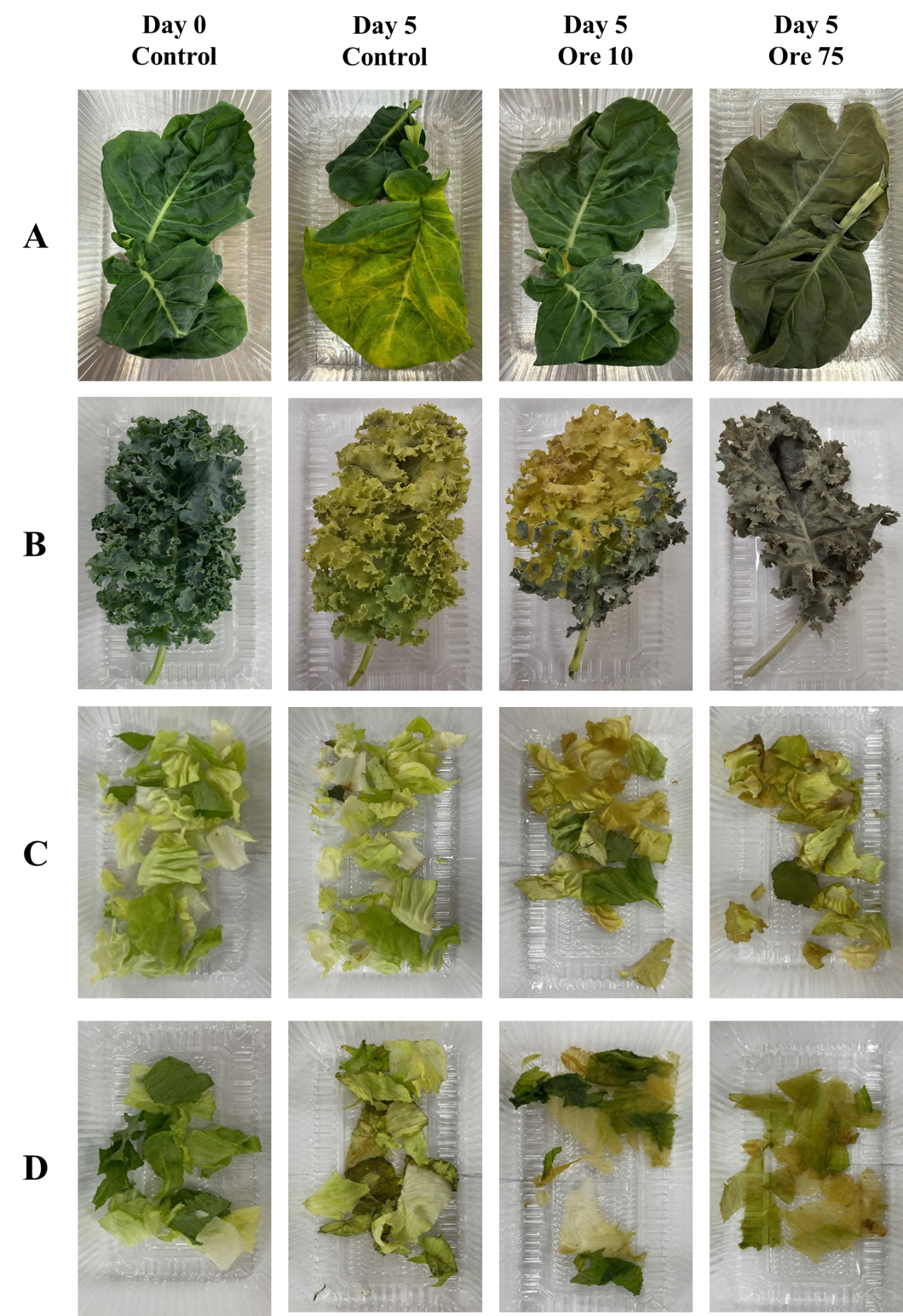


**Fig. S3.** Screening on vegetables suitable for the selected EO vapor treatment. (A) Kai Lan (B) kale (C) butter lettuce and (D) iceberg lettuce. (Notes: Control: no EO; Ore 10: 10 µL oregano EO vapor treatment; Ore 75: 75 µL oregano EO vapor treatment).
